# Towards Evaluating Pitch-Related Phonation Function in Speech Communication Using High-Density Surface Electromyography

**DOI:** 10.3389/fnins.2022.941594

**Published:** 2022-07-22

**Authors:** Mingxing Zhu, Xin Wang, Hanjie Deng, Yuchao He, Haoshi Zhang, Zhenzhen Liu, Shixiong Chen, Mingjiang Wang, Guanglin Li

**Affiliations:** ^1^School of Electronic and Information Engineering, Harbin Institute of Technology, Shenzhen, China; ^2^CAS Key Laboratory of Human-Machine Intelligence-Synergy Systems, Shenzhen Institute of Advanced Technology, Chinese Academy of Sciences, Shenzhen, China; ^3^Shenzhen College of Advanced Technology, University of Chinese Academy of Sciences, Shenzhen, China; ^4^Guangdong-Hong Kong-Macao Joint Laboratory of Human-Machine Intelligence-Synergy Systems, Shenzhen Institute of Advanced Technology, Chinese Academy of Sciences, Shenzhen, China; ^5^School of Instrument Science and Engineering, Southeast University, Nanjing, China; ^6^Surgery Division, Epilepsy Center, Shenzhen Children's Hospital, Shenzhen, China

**Keywords:** phonation function, pitches, speech communication, high-density, surface electromyogram

## Abstract

Pitch, as a sensation of the sound frequency, is a crucial attribute toward constructing a natural voice for communication. Producing intelligible sounds with normal pitches depend on substantive interdependencies among facial and neck muscles. Clarifying the interrelations between the pitches and the corresponding muscular activities would be helpful for evaluating the pitch-related phonating functions, which would play a significant role both in training pronunciation and in assessing dysphonia. In this study, the speech signals and the high-density surface electromyography (HD sEMG) signals were synchronously acquired when phonating [a:], [i:], and [ә:] vowels with increasing pitches, respectively. The HD sEMG energy maps were constructed based on the root mean square values to visualize spatiotemporal characteristics of facial and neck muscle activities. Normalized median frequency (nMF) and root-mean square (nRMS) were correspondingly extracted from the speech and sEMG recordings to quantitatively investigate the correlations between sound frequencies and myoelectric characteristics. The results showed that the frame-wise energy maps built from sEMG recordings presented that the muscle contraction strength increased monotonously across pitch-rising, with left-right symmetrical distribution for the face/neck. Furthermore, the nRMS increased at a similar rate to the nMF when there were rising pitches, and the two parameters had a significant correlation across different vowel tasks [(a:) (0.88 ± 0.04), (i:) (0.89 ± 0.04), and (ә:) (0.87 ± 0.05)]. These findings suggested the possibility of utilizing muscle contraction patterns as a reference for evaluating pitch-related phonation functions. The proposed method could open a new window for developing a clinical approach for assessing the muscular functions of dysphonia.

## Introduction

Phonation is an essential physiological process *via* which natural voices are produced to aid human communication during daily life activities (Alku, 2011). Fundamentally, a normal voice is characterized by loudness, pitch, and quality, among which pitch is considered the most significant contributor toward constructing intelligible sounds (Kryshtopava et al., 2017). A wide range of normal pitches can enhance vocalization with the rich capability to express one's interpersonal emotions for social communication (Kim et al., 2009; Craig et al., 2015). Pitches used to describe the sound on a scale from low to high are often characterized and quantified in terms of frequencies (in cycles per second or hertz) (Rinaldi et al., 2016). For instance, to deliver a speech or sing a song with high intelligibility, the produced sound should consist of pitches accompanied by harmonic overtones that are multiples of the fundamental frequency (Bishop and Keating, 2012). However, an abnormal pitch resulting in a sound/vocalization that is too high, too low, unstable, monotonous, and unpleasant to listen would lead to phonating difficulty, often known as dysphonia (Jani and Gore, 2014; Knuijt et al., 2014; Sommerville et al., 2017). Also, dysphonia remains a major health issue, especially, for individuals whose jobs require high speech intelligibility such as teachers, singers, lawyers, tour guides, and salespeople, among others (Rosen and Murry, 2000; Cutiva et al., 2013; Martins et al., 2014). Therefore, early examinations and diagnoses of the pitch-related functions of dysphonia could be of great importance for these high-risk populations.

The evaluation of the normality of pitch-related phonation function plays a significant role in diagnosing dysphonia. Currently, most of the methods for assessing the pitch-related phonation functions are based on the analysis of speech signals (Gerratt et al., 2016; Brajot and Lawrence, 2018; Murtola et al., 2019). However, the speech signals are easily affected by environmental acoustic noises and human-introduced interferences (Dennis et al., 2010; Balata et al., 2015). It is difficult to collect speech signals with high quality in a noisy environment, and therefore, the recorded noise-contaminated speech signals were unreliable for assessing pitch-related phonation functions. Moreover, speech signals only contain one-dimension acoustic information about speech, and it is usually insufficient to evaluate the physiological process of phonation activities, the analysis of which requires the dynamic information of the related muscular activities.

Notably, phonation is a complex process controlled by multiple articulatory muscles (Chhetri and Neubauer, 2015). The construction of a normal pitch relies on the oscillation rate of the vocal cords controlled by the contraction patterns of the inherent and extrinsic articulation of the facial and neck muscles associated with phonation (Horáček et al., 2016). The strength, contraction rate, and coordination of the facial and neck muscles are essential factors in the construction of normal pitches during phonation (Macdonald et al., 2012). Therefore, examining the muscular activities during pitch-related phonation tasks is crucial for evaluating the phonating function. It is noteworthy that surface electromyography (sEMG) is an important technique for detecting, recording, and interpreting the electrophysiological characteristics of muscular activities (Tang et al., 2018). Meanwhile, the sEMG approach is non-invasive, safe, easy to operate, and cost-effective when compared with other methods (Naik et al., 2015; Strazzulla et al., 2016), making it widely utilized for assessing the muscular function associated with phonation activities (Pettersen et al., 2005; Van Houtte et al., 2013; Khoddami et al., 2017; Kaneko et al., 2018; Xu et al., 2018). For instance, four channels of sEMG signals recorded while the subjects were phonating a set of vowels at an increasing pitch were utilized to study the electrical activities of scalenus, sternocleidomastoideus, and upper trapezius muscles (Pettersen et al., 2005). In another study, time-domain features of sEMG signals from two channels were used to assess the functions of the cricothyroid and thyrohyoid muscles in patients with dysphonia (Khoddami et al., 2017).

Up to date, most of the previous studies mainly focused on assessing pitch-related phonation functions individually using either speech signals (Gerratt et al., 2016; Brajot and Lawrence, 2018; Murtola et al., 2019) or sEMG recordings (Pettersen et al., 2005; Van Houtte et al., 2013; Khoddami et al., 2017; Kaneko et al., 2018; Xu et al., 2018), whereas the quantitative interrelationship between the pitches and the corresponding muscular activities during the pitch-related phonation remains unclear. It is noteworthy that the interrelations between the pitches and the corresponding muscular activities are an essential prerequisite for exploring the electrophysiological mechanisms of pitch-related phonation functions, and such mechanisms play an important role in pronunciation training and dysphonia diagnosing. Therefore, the synchronous analysis of both speech signals and sEMG recordings is necessary to clarify the interrelations between the pitches and muscular activities.

Furthermore, since the phonation process involves a large group of small facial and neck muscles that span a relatively large area (Dewan et al., 2017), dynamically evaluating the electrophysiological spatiotemporal properties of the entire group of coordinated facial and neck muscles could be helpful for the analysis of the pitch-related phonation functions. However, most of the existing studies utilized only a few surface electrodes (typical 2–4 channels) with limited coverage, and they rarely provided adequate neuromuscular information required for consistently accurate diagnoses (Pettersen et al., 2005; Khoddami et al., 2017). A small number of sEMG electrodes might miss important muscles and major electrical activities that would be essential for the dynamic assessment of the entire phonation process. Therefore, a large number of sEMG electrodes would be necessary to cover the whole group of facial and neck muscles in enough density so that the spatiotemporal properties and coordination activities of all the muscles could be studied systemically.

High-density sEMG (HD sEMG) is a non-invasive technique to measure sEMG signals with a two-dimension array of closely spaced electrodes, and it could provide comprehensive information for dynamic evaluation of the spatiotemporal characteristics of muscle groups (Johns et al., 2016; Zhu et al., 2017b, 2018b; Chen et al., 2018; Glaser and Holobar, 2018). When applied in the phonation study, the HD sEMG technique could be beneficial for collecting sufficiently relevant information to evaluate the properties of the facial and neck muscles across the entire phonation process. In this regard, a series of sequential energy maps constructed from HD sEMG recordings could be utilized to evaluate the muscular activities of the frontal neck while phonating vowel [a:] in our pilot studies (Zhu et al., 2017a, 2018a). Afterward, Bracken et al. utilized a 20-channel array of HD sEMG signals located around the anterior neck to evaluate the muscular activities when phonating in three manners (rest, low, and high pitches) (Bracken et al., 2019). Nevertheless, these studies only focused on myoelectric characteristics of the neck muscles without simultaneously considering the properties of speech signals that are commonly applied in clinical settings, and therefore, the interrelations between the speech signals and the sEMG recordings during pitch-related phonation have rarely been investigated.

In this study, we investigated the possibility of utilizing the acquired dual-signal (speech and HD sEMG signals) for systematically exploring the interrelations between the pitches (sound frequencies) and the corresponding muscular activities associated with pitch-related phonation. This purpose had been achieved by dynamically visualizing the electrophysiological spatiotemporal properties of the facial/neck muscles and quantitatively analyzing the correlations between sound frequencies and myoelectric characteristics when phonating with increasing pitches across different vowel tasks. The proposed method might open a new window for developing a clinically relevant approach for training the pronunciation and diagnosing the dysphonia.

## Methods

### Subjects

Toward reducing the complexity of the subjects, a total of 14 healthy male volunteers (mean age = 24.7 ± 1.5 years) participated in this study involving a set of systematically designed phonation tasks. Before the experiments, all the subjects were enquired to ensure that they had no history of phonation difficulties, and a pure-tone audiometry test was conducted to ensure that they had no hearing problems. Then, the objective and experimental procedures of the experiments were clearly explained before the data collection session. All the subjects gave written informed consent and provided permission to publish their photographs/data for scientific and educational purposes. The protocol of this study was approved by the Institutional Review Board of Shenzhen Institutes of Advanced Technology (#IRB ID: SIAT-IRB-170815-H0178).

### Experimental Procedures

**Figure 1:**
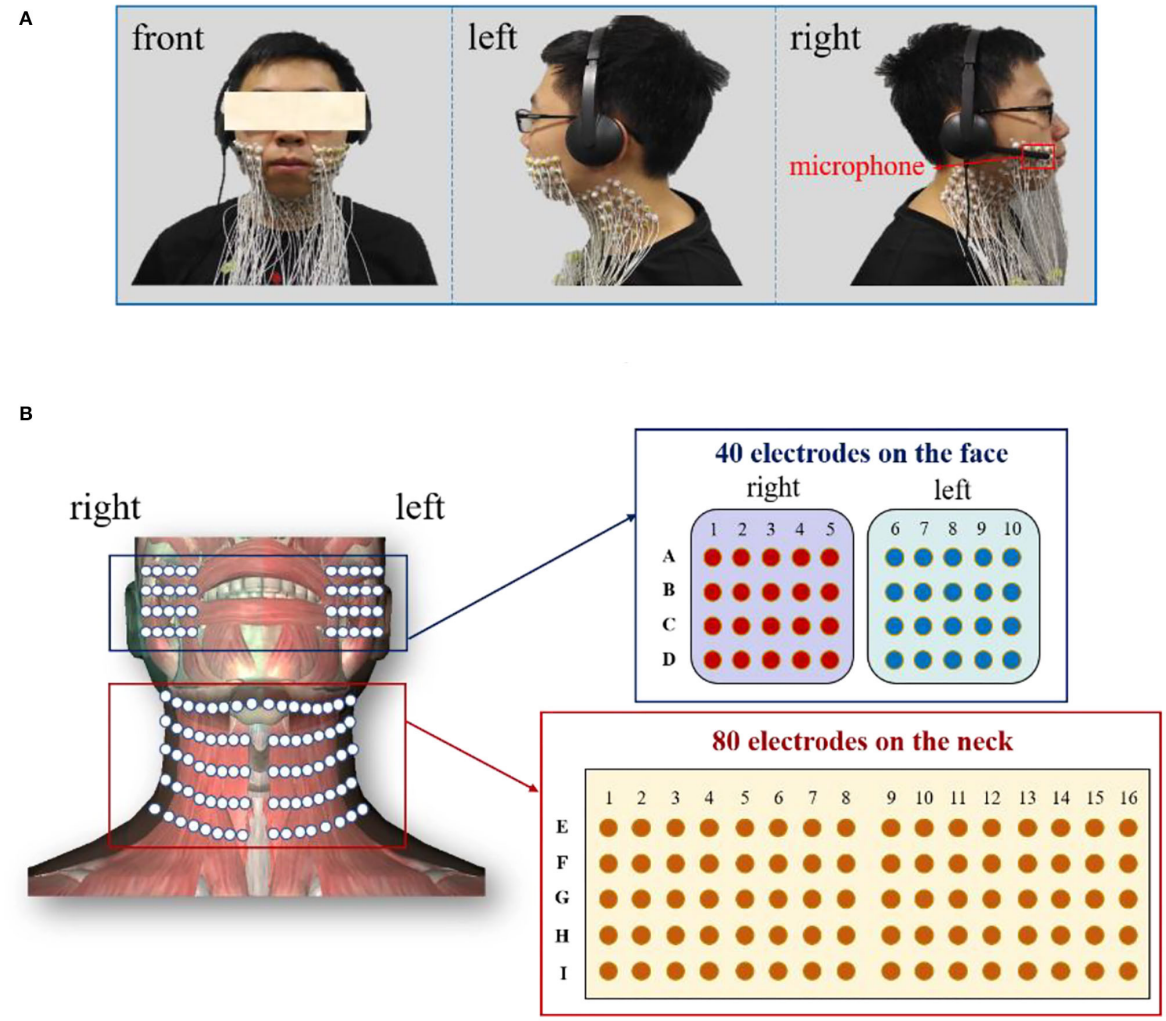
**(A)** Experimental setup to record the speech and HD sEMG signals simultaneously; **(B)** Placement of the high-density surface electrodes on the facial and neck muscles.

In this study, the speech signals and the HD sEMG recordings were simultaneously acquired from all the recruited subjects when a subject phonated the back vowels [a:], front vowels [i:], and central vowels [ә:] with a continuously increased pitch. The speech signals were recorded at a sampling rate of 44,100 Hz *via* a headset-attached microphone placed above the first row of sEMG electrodes on the right side of the face, as seen in Figure 1A. Meanwhile, the HD sEMG signals were recorded by using a multichannel EMG recording system (TMSI, REFA, the Netherlands) at a sampling rate of 2,048 Hz. During the experiment, we implemented a hardware circuitry that could trigger the start of the data acquisition of both the speech and HD sEMG to achieve the synchronization of the two types of signals. By using double-sided small-sized tapes, a total of 120 channels of surface electrodes were evenly placed on the skin surface of the face and the neck, both with left/right symmetry (Figure 1B). The double-sided tapes were medical grade and had very strong adhesive strength to stick the electrode on the skin to avoid the interferences introduced by the electrode wire movements or lip motion. Before placing each electrode, the skin of the subject was carefully prepared with an abrasive skin preparation gel and then cleaned with alcohol cotton to remove any sweat and oil that could possibly reduce the adhesion of the double-sided tapes.

Each surface electrode is ~10 mm in diameter, and an inter-electrode distance of about 15 mm was ensured between two neighboring electrode centers to cover the phonation-related muscles in intensive densities. In this regard, a two-dimension (2D) array made up of 80 electrodes in a 5 × 16 grid was placed on the suprahyoid and infrahyoid muscles located in the front neck regions of each subject, as seen in Figure 1B. The electrodes were identified and labeled by 5 rows (E–I) and 16 columns (1–16). The remaining 40 electrodes were symmetrically placed over the facial muscles (including the masseter, the buccinators, the orbicularis oris, and the zygomatic minor muscles), with a 4 × 5 grid on both the left and right sides. The 2D facial electrodes were identified and labeled by 4 rows (A–D) and 10 columns (1–10), as seen in Figure 1B. The extra space in the middle between the left and right neck electrode arrays was to avoid the laryngeal prominence of the subjects, especially in male subjects. Afterward, a fabric electrode was placed on the left wrist of the subject to serve as the reference electrode.

The phonation experiments were individually carried out on all the recruited subjects in an electromagnetic-shielded and soundproofed room that allowed the collection of high-quality electric and acoustic signals. Three different phonating experiments were adopted in the current study to assess the muscle activation patterns and to explore the interrelations between the speech and HD sEMG signals. First, the subjects were asked to maintain a quiet state without phonating or moving their body parts for around 30 s so that the baseline for the speech and the HD sEMG signals could be determined. Then, the subjects were asked to pronounce [a:], [i:], and [ә:] vowels, respectively, in a sequential order with a monotonously rising pitch (from low to high) for three successive repetitions per trial. In each repetition, the target vowel was phonated for ~4 s and followed by a rest period of 6 s that comprised 3 s before the phonation and 3 s after the phonation to prevent the subjects from experiencing either muscle or mental fatigue that may compromise the fidelity of the signals. During the phonation, the speech signals and the HD sEMG recordings were synchronously acquired from all the subjects.

### Analysis of the Speech Signals

The spectrogram of the speech signals during the phonation task was derived by using the short-time Fourier transform (STFT) method to perform the time-frequency analysis. The duration of the STFT analysis window was 20 ms, and the overlap width between two neighboring windows was 10 ms. The formula of the STFT method implemented in this study could be expressed as follows:


(1) $$\begin{array}{r} \text{STFT}\left\{x\left[n\right]\right\}\left(m,\omega \right)=X\left(m,\omega \right)=\\ \;\overset{\infty }{\underset{n=-\infty }{\sum }}x\left[n\right]\omega \left[n-m\right]e^{-j\omega n} \end{array}$$

where x[n] is the segmental speech signal at time frame n and ω[n-m] is a Hamming window with the same duration as x[n].

To further illustrate the frequency domain features, the speech signals obtained during the phonation tasks were segmented into a series of frames with a duration of 250 ms, and the amplitude spectrum of each frame was calculated accordingly. Then, frame-by-frame spectral curves could be obtained to demonstrate the frequency-domain characteristics of the speech signals when the subject was phonating vowels in an increasing pitch. Afterward, the median frequency (MF) of the speech signals from all frames was computed. MF is defined as the frequency at which the spectrum is divided into two regions with equal amplitude; in other words, MF is the frequency at half of the power spectral density of the signals (Phinyomark et al., 2012). It can be expressed as given in Equation (2). Finally, the MF values were normalized by employing the min-max normalization method given in Equation (3).


(2) $$\begin{array}{r} \overset{MF}{\underset{j=1}{\sum }}p_{j}=\overset{M}{\underset{j=MF}{\sum }}P_{j}=\frac{1}{2}\overset{M}{\underset{j=1}{\sum }}P_{j} \end{array}$$

where *P*_*j*_ is the EMG power spectrum at frequency bin *j* and *M* is the length of the frequency bin.


(3) $$\begin{array}{r} nMF\left(i\right)=\frac{MF\left(i\right)-\min\left(MF\right)}{\max\left(MF\right)-\min\left(MF\right)} \end{array}$$

where nMF(i) is the normalized MF value of speech signals, *MF(i)* is the MF value in analysis window *i*, min(MF) is the minimum MF value, and max(MF) is the maximum MF value during the entire phonation.

### Analysis of the HD sEMG Signals

A set of digital filters were used to enhance the quality of the HD sEMG signals obtained during the different phonation tasks. In this regard, we first used a third order Butterworth band-pass filter from 10 to 500 Hz to reduce baseline fluctuations. Then, a Butterworth band-stop filter was also applied to attenuate the power line interferences at 50 Hz and its harmonics. After the pre-processing, the filtered sEMG signal of each channel was sliced into a series of sequential 250 ms analysis windows, and the root-mean square (RMS) was computed for each analysis window to obtain the average energy distribution of the muscular activities as follows:


(4) $$\begin{array}{r} \text{RMS}\left\{sEMG\left[n\right]\right\}=\sqrt{\frac{1}{n}\overset{n}{\underset{i=1}{\sum }}sEMG^{2}\left[i\right]} \end{array}$$

where RMS{*sEMG*[*n*]} is the RMS value of sEMG signals for each analysis window, *sEMG*[*i*] is the *i*th sample in the analysis window, and *n* is the total sample number of the analysis window.

Then, the frame-by-frame RMS values of all the 120 channels were computed to investigate the dynamic activities of the facial and neck muscles during the phonation with continuously increasing pitches. Besides, RMS values of the same time frame from different neighboring channels were joined together to form a 2D array according to the electrode positions so that a 2D energy map could be obtained to present the energy distribution of the corresponding region. In this way, an energy map with a size of 4 × 5 could be obtained for the left and right faces, respectively. A 5 × 16 energy map could also be generated for the neck region. Then, the energy maps of all the time frames were globally normalized for the face and neck region, respectively, so that the sequential energy maps in a frame-wise manner could help to demonstrate the spatial and temporal changes in energy and muscular activities during different phonation stages.


(5) $$\begin{array}{r} nRMS\left(i\right)=\frac{RMS\left(i,j\right)-\min\left[RMS\left(i\right)\right]}{\max\left[RMS\left(i\right)\right]-\min\left[RMS\left(i\right)\right]} \end{array}$$

where nRMS(i) is the normalized RMS value of sEMG signals in channel *i, RMS*(*i, j*) is the RMS value of channel *i* in analysis window *j*, min[RMS(i)] is the minimum RMS value of channel *i*, and max[*RMS*(*i*)] is the maximum RMS value of channel *i*.

### Correlation Analysis Between Speech and sEMG Signals

The nMF of the speech signals, as well as nRMS values of the HD sEMG signals per analysis window, was obtained and compared to investigate the correlations between the speech signals and muscular activities. The entire phonation process with a duration of 10 s was segmented into a series of analysis windows with a length of 250 ms, resulting in a total of 40 nMF and 40 nRMS values. Then, the correlation coefficients between the two groups of nMF and nRMS were calculated to analyze the interrelations between the speech and sEMG signals.

## Results

### Features of the Speech Signals With an Increasing Pitch

**Figure 2:**
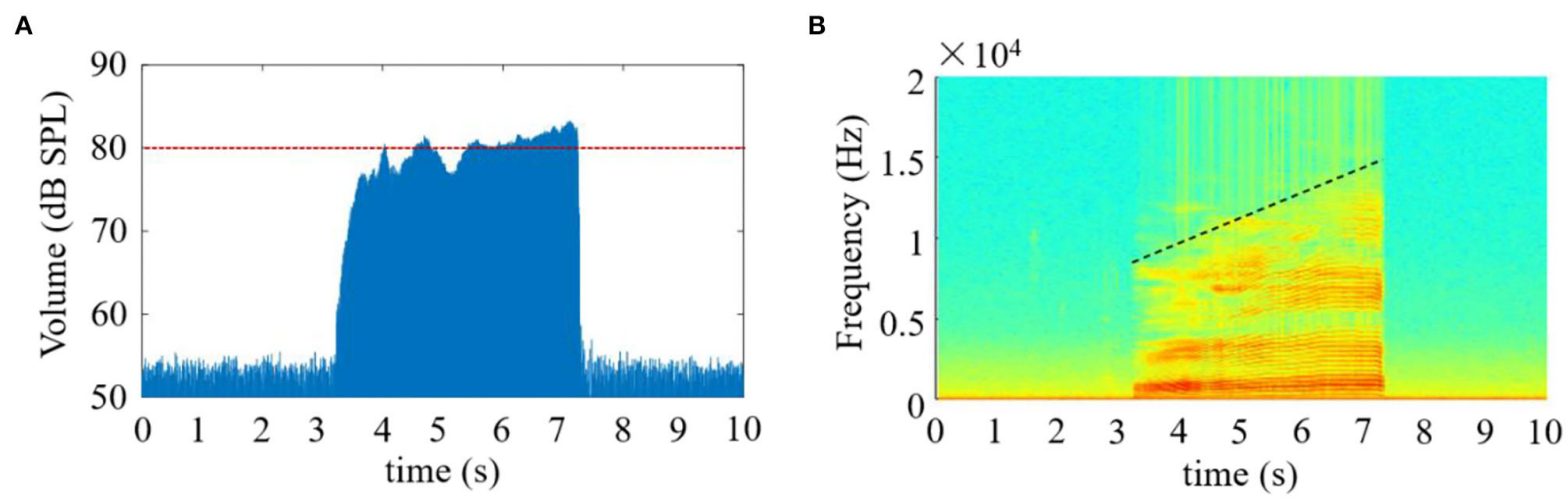
The amplitude and spectrogram of the speech signals when phonating the vowel [a:] with continuously increasing pitch. **(A)** The amplitude of the speech signal as a function of time; **(B)** The time-frequency spectrogram of the speech signal.

In this study, the time-domain and time-frequency characteristics of the speech signals obtained during the phonation process of [a:] vowel using low to high pitch were analyzed as follows. The waveform characteristic of the speech signals in the time-domain is seen in Figure 2A. From this figure, the speech signals were observed to immediately rise to ~76 dB SPL (calibrated through a professional sound level calibrator, Model AWA6021A, Gester Instruments Co., China) at the commencement of the phonation, and about 4 s later, the volume of the signal fluctuated around 80 dB SPL, thus exhibiting a relatively stable phenomenon when the pitch increased continuously from low to high. The speech signals instantaneously returned to the baseline at the completion of the phonation task.

Meanwhile, the time-frequency characteristics of the speech signals along the time axis are presented in Figure 2B. According to the analysis herein, the time-frequency distribution during phonation revealed that the high intensity of the speech signals was obtained from low-frequency components, especially between 0 and 8 KHz, which covered the entire phonating activities. Additionally, the frequency values of the speech signals were observed to increase steadily with a corresponding rise in pitch when [a:] vowel was phonated. Also, the highest frequency components appeared at the end of the phonation, where the pitch also got to its peak.

**Figure 3:**
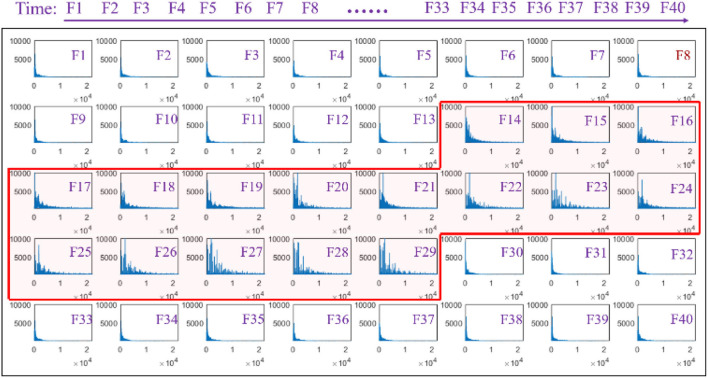
Amplitude spectra of the speech signal with an increasing pitch from a series of analysis windows (F1–F40).

A series of waveforms across the phonation process of [a:] vowel was constructed to characterize the amplitude spectrum of the speech signals. In this regard, the speech signals were segmented into 40 continuous windows of a length of 250 ms, otherwise known as frames from which the amplitude spectrum waveforms were later obtained and presented in a successive manner, as seen in Figure 3. Each spectrum showed the amplitude of each individual frequency within the corresponding time frame of speech to visualize the dynamic variation in the frequency domain. The amplitude spectrum of the frames (F1–F13) that correspond to the speech signals obtained before phonating the vowel [(a:)] was observed to have a low-frequency band with low intensities. Meanwhile, the amplitude spectra were observed to vary considerably between the 14th and 29th frames when the subjects began phonating at a low pitch and steadily increased to a high pitch. Specifically, at the beginning of the phonation task represented in frames F14–F16, the frequency band of the speech signals increased slightly to values below 10 KHz with a corresponding increase in the amplitude of the frequency. With a further increase in the pitches, the frequency band of the signals became even wider from frames F17–F29 with a band that was ~20 KHz. Meanwhile, the amplitude of the frequency also increased steadily from F17–F29 with a corresponding increase in pitches. Subsequently, the frequency band and the amplitude of the speech signals suddenly dropped when the subjects stopped phonating.

### Temporal Waveforms of the HD sEMG Signals Across the Phonation Process of [a:] Vowel With a Rise in Pitch

**Figure 4:**
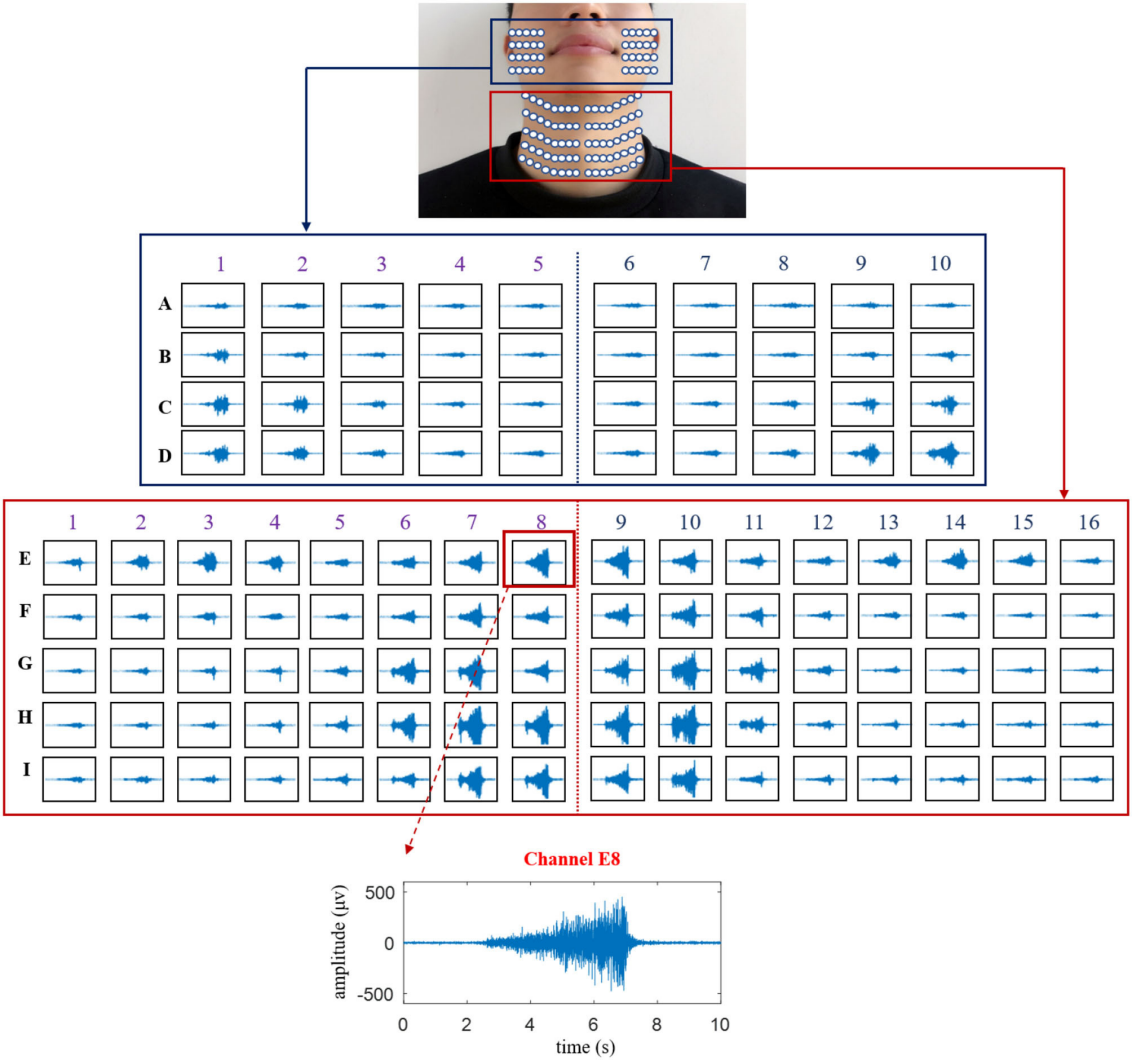
The temporal waveforms of the HD sEMG signals recorded from all the 120 surface electrodes on the facial and neck muscles during the phonation with rising pitch.

The temporal waveforms of HD sEMG signals are displayed in Figure 4 for showing the characteristics of facial and neck muscles, in which the waveform of a typical channel (E8) was presented to exhibit the variation of the sEMG amplitudes. At the onset of the phonation task, the temporal waveforms of the HD sEMG signals associated with the facial and neck muscles had relatively lower intensity, and as the pitch increases from low to high, the amplitude of the waveforms also increased correspondingly across all the channels. Meanwhile, at the offset of the phonation task, the HD sEMG waveforms were observed to have a sudden drop in amplitude to the baseline.

As seen in Figure 4, the HD sEMG waveforms were constructed for 120 channels that were divided into two parts: the first denotes the facial region (in the blue rectangle) consisting of 40 channels (from columns 1 to 10 and rows A to D) and the other part indicates the neck region (in red rectangle) consisting of 80 channels (from columns 1 to 16 and rows E to I). On the facial region (row A to D), the amplitudes of the sEMG waveforms obtained from the electrodes located around the mouth (from columns 3 to 8) were relatively smaller than those obtained from the signals on the outer part of the face (right side: columns 1–2 and left side: columns 9–10) during the entire phonating process. On the contrary, as shown in the neck region (rows E–I), the amplitudes of the sEMG signals turned out to be larger at the center of the neck area (from columns 6 to 11) than those at the edge of the neck (right side: columns 1–5 and left side: columns 12–16). Meanwhile, the sEMG signals on the first row of the neck region (E1–E16) also showed high intensities when phonating with an increasing pitch. Additionally, the symmetrical distributions of the HD sEMG waveforms were observed between the left and right sides for both facial and neck regions.

### Dynamic HD sEMG Energy Maps When Phonating [a:] Vowel With Increasing Pitch

**Figure 5:**
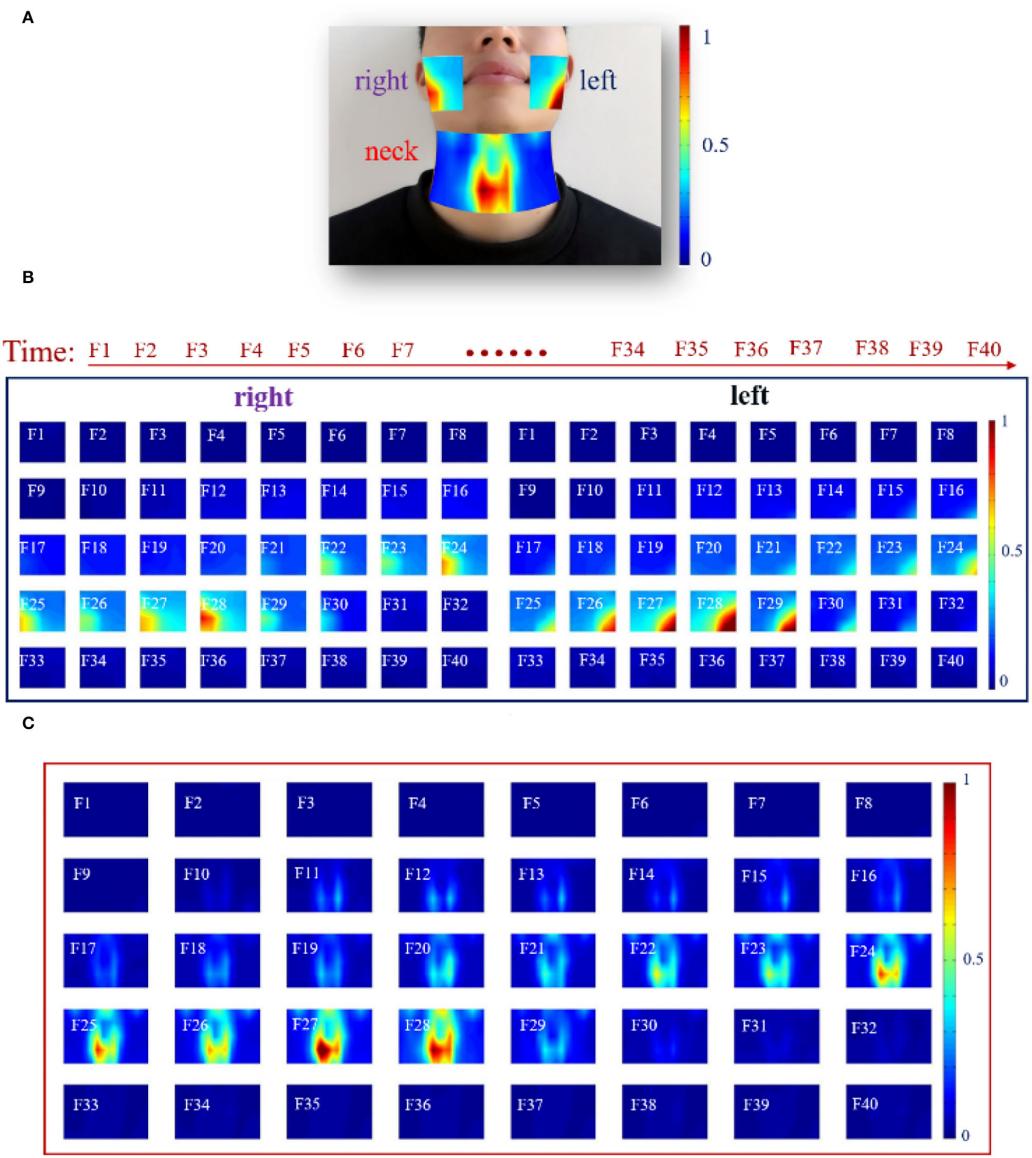
The HD sEMG energy maps of the facial and neck muscles by using 40 analysis windows with a length of 250 ms during phonating vowel [a:] with pitch increase: **(A)** A typical frame of HD sEMG energy map visualizing the facial and neck muscle activities; **(B)** HD energy maps of the facial muscles; **(C)** HD energy maps of the neck muscles.

In this section, dynamic HD energy maps were constructed to analyze the characteristics of the facial and neck muscles during the entire phonation process. For visualizing the dynamic changes in muscular activities associated with facial and neck muscles during rising pitches, the earlier described RMS feature was computed from a series of analysis windows that resulted in feature matrices utilized for generating the HD sEMG energy maps presented in Figure 5. Note that different intensities of myoelectric activities are represented by the color gradient of the energy maps, in which the color toward red signified high intensity and that toward blue denoted low intensity.

First, a typical dynamic HD sEMG energy map of the facial and neck muscles from an analysis window during the phonation process is displayed in Figure 5A. It could be seen that the map in the neck region had higher intensity, especially for muscles that appeared around the center of the neck. Thus, the muscles in the active neck region were distinctively separated into two parts symmetrically, in which the right side had slightly higher energy intensity than the left region of the neck. Meanwhile, low-intensity energy was observed on the top of the map that is symmetrical between the right and left sides. In addition, a highly symmetrical energy distribution was equally observed between the right and left sides. The energy map on the right side of the facial muscles had high intensity on the edge of the right face region, while the map on the left side of the facial muscles reflects high intensity on the left side of the facial muscles (Figure 5A).

Subsequently, a further analysis that involved the entire phonation process was done to observe if similar results could be obtained from a single channel described earlier. In this regard, the entire phonation task was segmented into 40 analysis windows of a length of 250 ms each, and a sequence of HD sEMG energy maps was constructed to reflect the dynamism of the myoelectric activities associated with the facial and neck muscles. Hence, a total of 40 frames of HD sEMG energy maps representing myoelectric activities of the facial muscles are shown in Figure 5B, while 40 frames of HD sEMG energy maps of the neck muscles are presented in Figure 5C.

At the onset of the phonation task (F1F10), the subjects were asked to assume a quiet state without moving. Hence, no activity could be seen for both the facial (Figure 5B) and neck (Figure 5C) muscles. Meanwhile, when the subjects began phonating, the HD sEMG energy maps became different between the facial and neck muscles, as could be seen from F11 to F30 in Figures 5B,C.

As seen in Figure 5B, the facial muscles were kept in unconspicuous activities on both the right and left sides from F11 to F20 even after the subject had already begun to increase the pitch. It was noticed that there was some delay in the facial muscle activation during the phonating process. Then, from F22 to F28, the intensity of the energy maps started constantly increasing to reach the maximum value on F28, and the high-intensity areas of the maps on the right and left sides were concentrated on the edge of the facial muscles. The active regions on the left side of the facial muscles appeared at the bottom left of the maps, while that on the right side of the facial muscles performed at the bottom right of the maps. Additionally, it was found that the HD sEMG energy maps of the facial muscles were symmetrically distributed on both the left and right sides, besides there were some differences between the two sides, in which the intensities of the left facial muscles were a little higher than that of the right facial muscles. After that, at the end of the phonating process, the myoelectric activities of the facial and neck muscles were diminished in a short time (from F29 to F30) when the subject prepared to stop pronouncing. Finally, after the subject went back to quiet, the high-intensity area of the energy maps disappeared from F31 to F40.

Then, it was observed that the muscular activities of the neck muscles (Figure 5C) were activated before the facial muscles (Figure 5B). The high intensity of the energy maps on the neck muscles started to emerge from the center of the map at the 11th frame in low intensity, while that of the facial muscles was activated at the 21st frame (Figure 5C). Afterward, the energy maps were maintaining a stable intensity along with the following five frames from F12 to F17. Later, from F18 to F28, the energy maps showed that the intensities in the center region of the neck muscles were gradually increased and spread upward to the upper locations when the subject was phonating vowel [a:] with continuous rising pitches. During the whole phonating process, the muscle activations were concentrated on the center of the neck muscles and split up into two parts by the centerline. Meanwhile, the energy maps clearly presented approximately symmetric activity regions on the left and right sides of the neck muscle. Also, the total intensity of myoelectric activities on both the right and left sides reached a maximum value at the 28th frame. Thereafter, at the end of the phonating process, the intensities of energy maps were diminished in the 29th frame in a short time. Finally, when the subject stopped phonating, the high-intensity area of the energy maps disappeared from F30 to F40 in Figure 5C. Besides, similar energy patterns of the dynamic HD sEMG maps on facial and neck muscles could also be observed in the other 14 subjects when they were phonating vowel [a:] with pitch increase.

### Interrelations Between Sound Frequencies and Myoelectric Activities During Phonating [a:] Vowel

**Figure 6:**
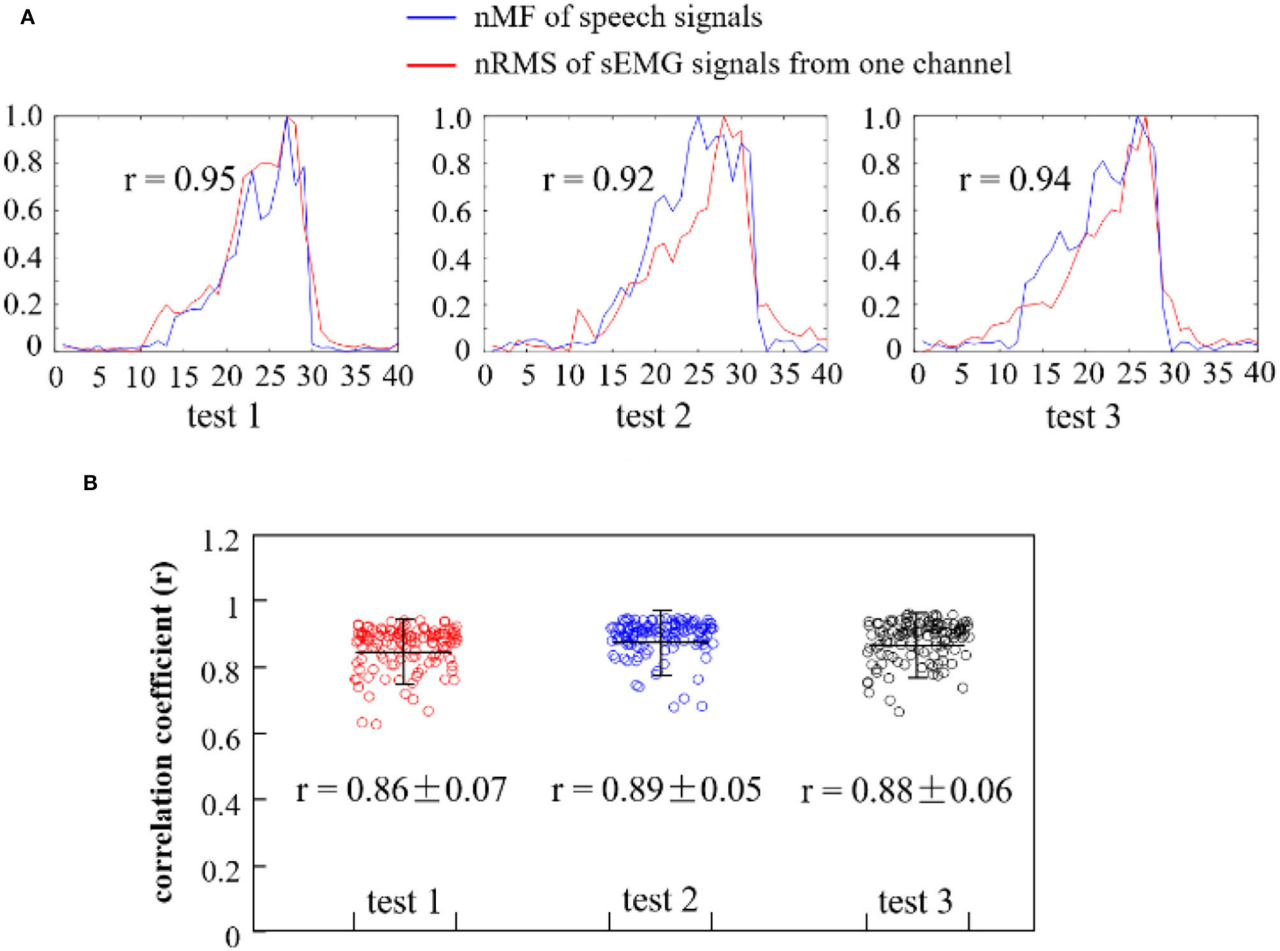
**(A)** Comparison of the nMF of the speech signals and the nRMS of the sEMG recordings from one channel when phonating vowel [a:] with increasing pitch; **(B)** Correlation coefficients between the nRMS values of 120 channels of sEMG recordings and the nMF of the speech signal from three repeated tests.

To quantitatively investigate the interrelations between the pitches of the speech signals and the intensity of the myoelectric activities while phonating vowel [a:] on a rising pitch scale, the nMF of speech signals and the nRMS values of sEMG recordings were compared across the entire phonating process. Then, the correlation coefficient between the nMF and nRMS features was computed across three repetitions of the phonation task, and the obtained result for a representative subject is presented in Figure 6.

In Figure 6A, the curves in blue showed the normalized nMF of the speech signals, while that in red represented the nRMS of the sEMG signals for a randomly selected channel. It could be observed that the waveforms of the nMF and nRMS features exhibited similar morphology in the first test session (test 1), in which the nRMS values (from sEMG signals) increased in a similar rate with the nMF values (from speech signals) at a constantly rising pitch. It could be seen that when the pitch reaches its maximum value, both the nMF and nRMS values rapidly declined in a similar manner until they both hit the baseline. This phenomenon is also observed for the other two test sessions (test 2 and test 3) in Figure 6A. The nRMS feature curve appeared before the nMF feature curve at the onset of the phonation task, while the nRMS feature curve disappeared after the nMF feature curve at the offset of the task. Furthermore, a significant correlation was observed between the nMF and the nRMS features (*r* = 0.95 in test 1, *r* = 0.92 in test 2, and *r* = 0.94 in test 3) at a *p* < 0.05 based on Pearson's correlation coefficient analysis. In other words, this analysis indicated that a strong correlation existed between sound frequencies and muscular activities associated with pitch-related phonation.

To further validate the above claim, the correlation coefficient between the nMF of the speech signals and the nRMS across the 120 channels of sEMG recordings in the three repeated tests was computed, and the obtained result is presented in the scatter plots seen in Figure 6B. The red, blue, and black scatterplots separately showed the correlation coefficients corresponding to test 1, test 2, and test 3. The correlation coefficients were closely clustered with an average value of 0.86 in test 1, 0.89 in test 2, and 0.88 in test 3 for a representative subject, indicating that there was a significant correlation between the nMF of the speech signals and the nRMS of the sEMG signals across the three repeated tests. Additionally, a few points were seen to be dispersed away from the center of the cluster, which could be a result of either random noises or unintentional body movements that must have occurred during the data acquisition session.

### Averaged Dynamic HD sEMG Energy Maps When Phonating [i:] and [ә:] Vowels on a Rising Pitch Scale

**Figure 7:**
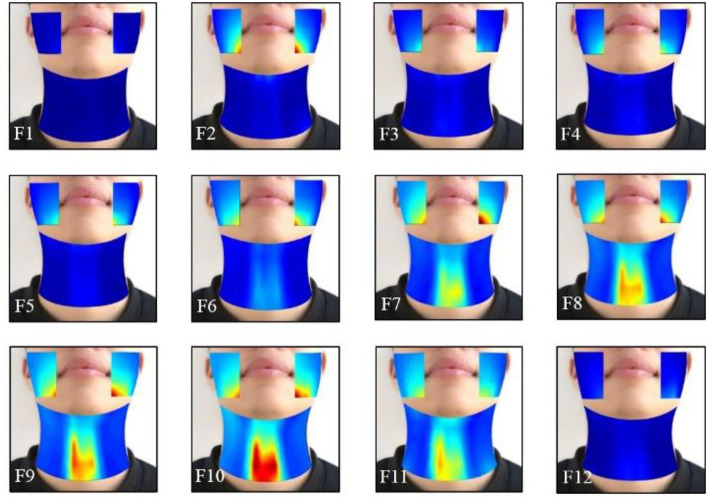
The averaged HD sEMG energy maps of the facial and neck muscles during the phonating vowel [i:] with a pitch increase.

**Figure 8:**
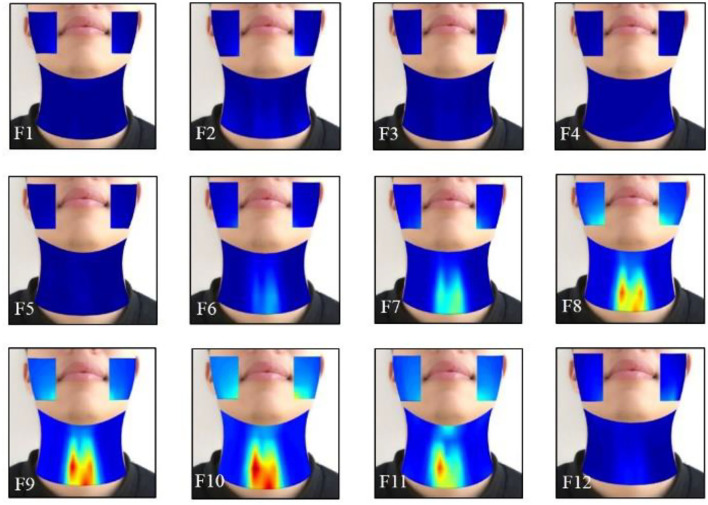
The averaged HD sEMG energy maps of the facial and neck muscles during the phonating vowel [ә:] on a rising pitch scale.

The effects of the facial and neck muscle activities across different vowels ([i:] and [ә:]) during the normal phonating processes were also investigated using the HD sEMG energy maps from another subject. Each phonating task that contained 40 frames of energy maps was truncated to 24 frames (from frame 9 to 32). Then, two of the adjacent frames from the remaining 24 maps were averaged with non-overlapped epochs to construct 12 consecutive energy maps (F1–F12). It can be seen from Figures 7, 8 that the HD sEMG energy maps from the two phonating tasks showed the explicit difference with regard to the duration and strength of the facial and neck muscle contractions.

Figure 7 showed the averaged dynamic sEMG energy maps when one subject was phonating [i:] vowel. When starting phonating, the high-intensity areas were first observed on the sEMG maps of the facial muscles (from F2 to F5), while the activities of the neck muscles were kept at low intensity. There was some delay in the neck muscle activation than the facial muscles during the phonating process. Then, from F6 to F10, the intensity on the energy maps of both the facial and neck muscles started increasing continuously to reach the maximum value on F10. The high-intensity areas of facial muscles were concentrated below the angulus oris on the right and left sides, while the areas were over the center of the neck muscles with high symmetrical distribution on the left and right sides of the neck. Meanwhile, the activated areas of the facial and neck muscles were expanded during increasing pitches (F6–F10). After that, the intensity of the maps was diminished in a short time at frame 11 when the subject prepared to stop phonating. Finally, the high-intensity area of the energy maps disappeared in frame 12 at the end of the phonating tasks.

The HD sEMG energy maps in Figure 8 represent a sample of the energy distribution of the facial and neck muscles when phonating the vowel [ә:] on a rising pitch scale, which was observed to be consistent in the other 13 subjects. Before the phonation, the maps showed a low intensity from the 1st through the 5th frame. Then, the intensities of the energy maps on the neck muscles were gradually increased from frame 6 to 10, while the maximum intensity was achieved in frame 10. The areas of the high intensity were also gradually expanded with the symmetrical distribution of both the left and right sides. Meanwhile, the high light areas of the map on the facial muscles first appeared at the bottom edge of the maps in low intensity (in F8), where the activation time was delayed than the neck muscles. After that, the intensities on the maps were increased until F10 on both the left and right sides of the face and the highlighted areas were enlarged when increasing pitches. Then, the intensity of the energy maps on the facial areas reduced at F11 and finally disappeared at F12 after the subject stopped phonating (Figure 8). The intensities of the neck muscles were higher than that of the neck muscles, which revealed that the neck muscles were more active than the facial muscles when phonating [ә:].

### Correlations Between the nMF of the Speech Signals and the nRMS of the sEMG Recordings Across Different Vowel Tasks

**Figure 9:**
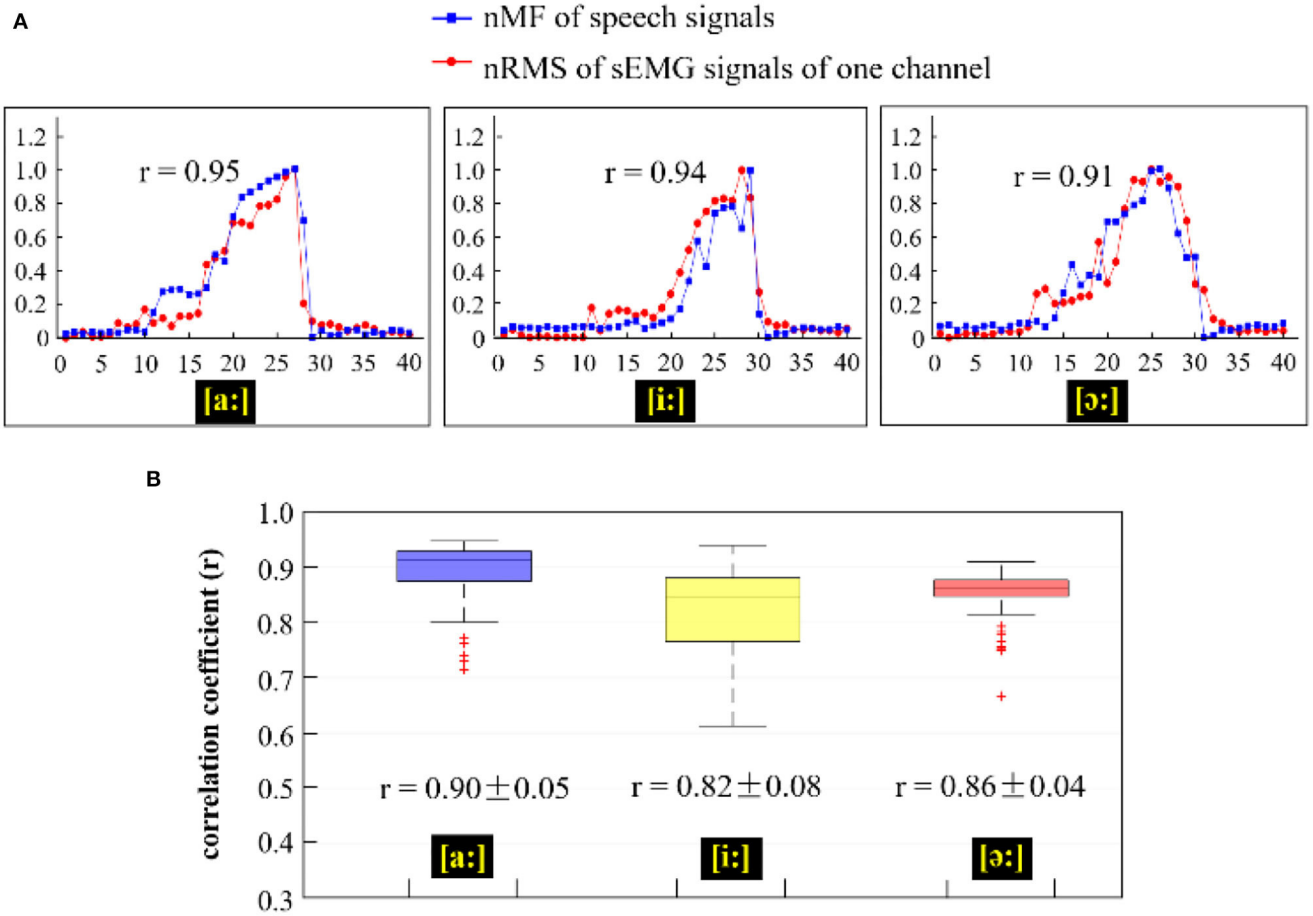
**(A)** Comparison of the nMF of the speech signals and the nRMS of the sEMG recordings from channel E8 when phonating three different vowels [(a:), (i:), and (ә:)] with increasing pitch; **(B)** Correlation coefficients between the nRMS values of 120 channels of sEMG recordings and the nMF of the speech signal across three different vowels [(a:), (i:), and (ә:)].

In this section, the correlations between the nMF of the speech signals and the nRMS of the sEMG recordings were investigated for the three phonating tasks including the back [(a:)], front [(i:)], and central [(ә:)] vowels across subjects when the pitch increased from low to high, and the obtained results are shown in Figure 9 and Table 1.

**Table 1:** The average correlation coefficients between the nRMS of sEMG recordings from all channels and the nMF of the speech signals across all recruited subjects (*p* < 0.05).

	**Sub**	**1**	**2**	**3**	**4**	**5**	**6**	**7**	**8**	**9**	**10**	**11**	**12**	**13**	**14**
[a:]	M	0.83	0.94	0.85	0.94	0.83	0.86	0.90	0.87	0.87	0.83	0.89	0.95	0.90	0.89
	SD	0.02	0.02	0.04	0.02	0.06	0.02	0.03	0.1	0.04	0.03	0.03	0.01	0.03	0.01
	0.88 ± 0.04														
[i:]	M	0.88	0.90	0.91	0.96	0.86	0.84	0.82	0.92	0.83	0.90	0.91	0.92	0.88	0.90
	SD	0.05	0.02	0.02	0.02	0.05	0.03	0.07	0.02	0.03	0.03	0.06	0.03	0.03	0.05
	0.89 ± 0.04														
[ә:]	M	0.90	0.92	0.82	0.95	0.90	0.87	0.78	0.87	0.90	0.89	0.79	0.95	0.81	0.88
	SD	0.02	0.01	0.05	0.01	0.01	0.01	0.02	0.03	0.04	0.08	0.03	0.01	0.08	0.04
	0.87 ± 0.05														

In Figure 9, the nRMS values from one channel of sEMG (red lines) and the nMF values (blue lines) from the speech signals for a specific subject were examined and compared across the three different phonating tasks [(a:), (i:), and (ә:) vowels]. From Figure 9A, it could be seen that the nMF of the speech signals was gradually rising when the phonating vowel [a:] was with a constantly increasing pitch and suddenly drop to the baseline after the maximum pitch is attained, indicating the end of the phonation task. Meanwhile, the nRMS of the sEMG recordings also exhibited similar characteristics as the nMF at the onset, intermediate, and offset points. However, the curve of the nRMS got initiated before that of the nMF at the onset of the phonation task and as well as hit the baseline after the nMF curve at the offset of the task. In like manner, the phenomenon observed between nMF and nRMS while phonating the [a:] vowel was also observed when phonating [i:] vowel and [ә:] vowel. In summary, significantly high correlations were recorded between the nMF of the speech signals and the nRMS of the sEMG signals during the three phonation tasks: [a:] (*r* = 0.95, *p* < 0.05), [i:] (*r* = 0.94, *p* < 0.05), and [ә:] (*r* = 0.91, *p* < 0.05).

Moreover, to investigate the interrelations between the nMF of the speech signals and the nRMS from the 120 channels of sEMG recordings across different vowel tasks, the correlation coefficient of the two parameters was calculated and displayed as box plots shown in Figure 9B. The blue-violet, yellow, and red boxes in Figure 9B show the correlation coefficients corresponding to phonating [a:], [i:], and [ә:] vowels, respectively. The correlation coefficients had overall average values of 0.90 ± 0.05 in [a:] vowel task, 0.82 ± 0.08 in [i:] vowel task, and 0.86 ± 0.04 in [ә:] vowel task, which revealed that there was a significant correlation between the nMF of the speech signals and the nRMS of the sEMG signals across the different phonating tasks. Additionally, a few overflows were observed to be dispersed away from the central areas in phonating [a:] and [ә:] vowels.

To further confirm if the correlations between the speech signals and the sEMG recordings are consistent across all of the individuals, the correlation coefficients between the nMF and nRMS values for all the 14 subjects were obtained when [a:], [i:], and [ә:] vowels were phonated from a low to a high pitch (Table 1). The results presented in Table 1 are expressed in terms of the mean and standard deviation values of the correlation coefficients across the three repeated tests for all 14 subjects. It could be observed from Table 1 that for the [a:] vowel, an average correlation coefficient of 0.88 ± 0.04 (*p* < 0.05), was achieved across subjects while a maximum correlations value of 0.95 and was recorded by subject 12, indicating a significant correlation between the nRMS and the nMF when phonating vowel [a:]. A mean correlation coefficient of ~0.89 ± 0.04 was recorded across subjects when the vowel [i:] was phonated, and of almost 0.87 ± 0.05 when phonating vowel [ә:], with 0.96 as the highest value (subject 4) also indicating significant interrelations between the nMF of the speech signals and the nRMS of HD sEMG recordings when phonating [i:] vowel with a continuous increase in pitch. Similarly, when the vowel [ә:] was phonated, an average correlation coefficient of 0.87 ± 0.05 was recorded across all participants with subject 5 attaining the maximum value (0.95) at *p* < 0.05, which also indicated significant correlations between the sound frequencies and muscular activities when the vowel [ә:] was phonated.

## Discussion

The principal objective of this study was to investigate the interrelations between the pitches and the corresponding muscular activities when phonating different vowels with increasing pitches, which would be helpful for evaluating the pitch-related phonating functions. This objective has been reached by dynamically visualizing the myoelectrical activities of the facial/neck muscles and quantitatively investigating the correlations between sound frequencies and myoelectric characteristics during different phonating tasks.

In this study, the speech and the HD sEMG signals were synchronously acquired from 14 subjects when phonating [a:], [i:], and [ә:] vowels with increasing pitches. The synchronous analysis of the speech and HD sEMG signals would clarify the interrelations between the pitches and the myoelectric activities of facial and neck muscles associated with pitch-related phonation. The sEMG signals display the potential difference between two separate electrodes above the muscles on the skin, which can be used to assess muscular function by recording electric potential activities generated by muscle cells that are from the inherent and extrinsic muscles. Moreover, phonating is a complex process controlled by multiple articulatory muscles, which contain the inherent and extrinsic muscles spanning a relatively large area of the face and neck. There were ~30 facial and neck muscles that are involved in a phonating movement. Therefore, a total of 120 channels of closely spaced electrodes were used to construct the 2D electrode arrays for recording the HD sEMG signals that were processed in the space dimension to assess global facial and neck muscle activities corresponding to pitch-related phonation. The use of the HD sEMG signals, which provided spatiotemporal information on muscular activities, could address the limitations that finite myoelectric information obtained from several muscles, which might not afford enough functional characteristics for dynamic assessment of the articulatory muscles associated with an entire phonation process. As it is known, the nMF of the signals is defined as the frequency value at which the signal power spectrum was divided into two sections of equal energy content, and it can afford an acceptably good representation of the sound frequency shift. Meanwhile, the nRMS values of the sEMG signals represent the average power of the myoelectric activities (Phinyomark et al., 2012). Therefore, the two parameters, nMF of the speech signals and the nRMS of the sEMG recordings, were extracted to quantitatively evaluate the correlations between sound frequencies and myoelectric features associated with pitch-related phonation.

During the phonating process, the characteristics of the speech signals demonstrated that the sound frequencies were increasing with the continuous rising of the pitches while the sound volume was kept on a stable scale (Figures 2, 3). These results indicated that the sound frequencies were tracked with the pitches, which was corresponding to the previous studies where the pitch was used to order sounds on a frequency-related scale (Lolli et al., 2015). In the current study, the results of the sound volume indicated that the effects on the muscle activities came from the variation of the sound frequency rather than the loudness.

Then, the temporal waveforms of HD sEMG signals across all the 120 channels on the facial and neck regions displayed that the myoelectric activities of the facial and neck muscles were orderly altered with the variation of the sound pitches (Figure 4). The sEMG waveforms from one electrode were different from each other even if it was from the two adjacent electrodes, which reveals that there was no cross-talk of the HD sEMG signals. The results suggested that the facial and neck muscles could produce synergetic muscle contractions to aid the movement of the passive and active articulators for constructing sound in high intelligibility with a wide scale of the pitch.

In this study, the typical normal phonating process (pitch rising) was divided into a sequence of duration, and the RMS values of the HD sEMG signals in each duration were calculated and visualized as pixels placed at a certain location of the electrode array on the facial and neck muscles. Thus, a series of 2D temporal sEMG energy maps of the facial and neck muscles were constructed for displaying the dynamic activities of the facial and neck muscles when phonating different vowels [(a:) in Figure 5, (i:) in Figure 7, and (ә:) in Figure 8]. Also, the spatial and temporal properties of the myoelectric activities were dynamically presented in the HD sEMG energy maps to visualize the muscular contraction patterns corresponding to the pitch-related phonation activities.

According to the HD sEMG energy maps, it was found that the facial and neck muscles were both activated during phonating tasks, and the strength of the facial and neck muscles gradually increased while the high-density areas of these muscles progressively enlarged when the pitches were continuously rising. These spatiotemporal properties of the facial and neck muscles corresponded to the physiological process of phonation as a transmission path of the airstream used for generating certain sounds, during which the airstream was expelled from the lungs through the throat (controlled by the neck muscles) into the mouth (controlled by the facial muscles) (Miller et al., 2009). Meanwhile, the sound pitches were determined by the oscillatory rates of the vocal cords with orderly opening and closing, which were controlled by the facial and neck muscles that were synergistically operated with alternate contracting and stretching in precision (Echternach et al., 2017). These coordinated sequences of the facial and neck muscles would produce myoelectric activities related to phonation activities. Moreover, when the sound pitches continuously increased from low to high, the vocal cords needed to vibrate faster and faster to reach the high pitches. During this pitch-increasing process, it could require more articulatory muscles and higher muscle strength to control the airstream transmitting faster for generating the faster vibration of the vocal cords, thus resulting in the higher intensities and larger areas of muscle activities on the face and neck. Additionally, the results of the energy maps also displayed that the intensities of the neck muscles were higher than that of the facial muscles, which reveals that the neck muscles would be the main contributors during phonating movements. The signals from the middle region of the neck demonstrated larger amplitude when compared with other regions, as could be observed from the columns 6–11 in Figure 4. When observing the energy distribution in Figure 5, similar consistent results were found for the activation patterns of the articulatory muscles with larger activities in the middle region of the neck in Figure 5A and middle frames in Figure 5B. This phenomenon is also based on the physiological characteristics of the phonating process, in which the vocal cords located inside of the larynx are the major organ to produce a natural voice, whose activities are controlled by the middle neck muscles.

Furthermore, the energy concentration was symmetrically distributed on the left and right sides of the facial and larynx regions, which indicated that the energy of muscles associated with phonation activities had a nearly equivalent intensity and durability on the right and left sides of the facial and neck muscles. Notably, there was a little difference between the left and right facial muscles, such as the intensities of the left facial muscles were a bit higher than that of the right one during phonation. This phenomenon would be based on personal habits in speaking and swallowing. For instance, if someone eats bolus by often using the left side of the teeth, the left facial muscles would be more exercised than the right one, which would lead to stronger muscles on the left face. Thus, when the subject phonated different vowels, the activities of the left facial muscles were higher than that of the right one. This result suggested that the HD sEMG energy maps might be a useful tool for guiding the subjects to rectify their undesirable habits in speaking and swallowing activities.

The HD energy maps obtained from different phonating tasks showed the effects of the facial and neck muscles across different vowels [(a:), (i:), and (ә:)]. Based on the HD energy maps, the high intensity of the maps on the neck region appeared before that of the facial areas, which indicated that the muscular activities from the neck muscles were activated before the facial muscles at the onset of the phonating [a:] and [ә:] vowels (Figures 5, 8). In contrast, the activities of the neck muscles presented a little more delay than the facial muscles when phonating the [i:] vowel (Figure 7). These results indicated that the styles of the pronunciation would produce different voices, which required sequential contractions of facial and neck muscles in a certain order. Moreover, the energy maps on the facial muscles showed that the high-intensity areas appeared on different muscles when phonating different vowels. For instance, when phonating back vowel [a:], the subject should first relax and open the mouth, while keeping the lips in a round shape; this manner of pronunciation would lead to the strong strength of the masseter muscles. Hence, the high intensities of the maps were concentrated on the edge of the face both on the left and right sides (Figure 5B). On the contrary, when phonating the front vowel [i:], the subject needed to extend the lips to the sides in a flat shape, which could cause the activation of the orbicularis oris muscles, resulting in the high-intensity activities around the mouth (Figure 7). Finally, when phonating the central vowel [i:], the mouth of the subject just needed to be opened slightly, and the lips should keep in relaxation. Thus, the strength of facial muscles was very weak, and there were low intensities presented in the energy maps of the facial regions (Figure 8). Moreover, no significant effects of the phonating task order on the HD sEMG results were found in our pilot experiments of this study.

Consequently, the dynamic properties of the HD sEMG energy maps were well corresponding to the physiological and biomechanical principles of phonation, which would make the HD sEMG method a simple and non-invasive tool for accurately visualizing the phonating process and assessing the temporal and spatial properties of facial and neck muscles associated with phonation functions. Moreover, it could also help researchers and laryngologists to better understand the dynamic muscular activities of the facial and neck muscles during phonation. The HD sEMG technique might pave the way for developing a clinically relevant approach to screen, diagnose the phonating disorder caused by muscle problems, and even provide a potential method to locate the abnormal muscles that lead to the phonating disorder. Furthermore, the findings from the proposed method are not only applicable to the assessment of phonating function but may also spur positive advancement in other rehabilitation areas, including swallowing function evaluation (Zhu et al., 2017b) and back pain rehabilitation (Hu et al., 2014), among others, that equally adopts sEMG maps.

Considering the fact that phonation is a complex process controlled by multiple articulatory muscles, the use of both speech signals and sEMG recordings are necessary indicators for the analysis of the pitch-related phonation activities (Zhu et al., 2017b; Murtola et al., 2019; Zhang et al., 2020). However, it should be noted that the correlations between the sound frequencies (pitches) and the muscular activities of facial and neck muscles during a pitch-related phonation remains unclear. In this investigation, the nMF of the speech signals and the nRMS of the HD sEMG recordings were measured and compared during a pitch increase across three different phonating tasks [(a:), (i:), and (ә:) vowels]. The results presented that the nRMS values (from sEMG signals) increased at a similar rate with the nMF values (from speech signals) when constantly increasing pitches (Figures 6, 9). These results demonstrated that a strong interrelation existed between the sound frequencies and the muscular activities associated with pitch-related phonation. Meanwhile, the results of the correlation coefficients showed that there were significant correlations between the nMF of speech signals and the nRMS of sEMG recordings across different vowel phonation tasks [back (a:), front (i:), and central (ә:)] (Table 1). The findings revealed that the muscular activity patterns of the facial and neck muscles related to phonation might be served as a reference for evaluating the pitch-related phonation functions.

The purpose of the present study focused on the normal phonating functions, so the patients with phonating disorders will be recruited for further study. The HD energy maps and the interrelations between the speech signals and the sEMG recordings obtained in this investigation might be used as the reference for further evaluating and diagnosing the phonating functions or dysphonia in clinical application.

## Conclusions

In this study, the speech and the HD sEMG signals were simultaneously measured when the subjects were phonating vowels with an increasing pitch. The sequential energy maps constructed from multichannel sEMG recordings showed that muscle contraction strength increased monotonously as the pitch increased, and left-right symmetrical distribution was observed for the face and neck regions when phonating different vowels with increasing pitch. The nRMS parameter of the HD sEMG signals increased in a similar pattern to the nMF parameter of the speech signals when the pitch rose, and there were significant correlations between the two parameters across different vowel tasks. The present study suggested that the muscle contraction patterns might be used as a reference for the evaluation of pitch-related phonation functions that is important for dysphonia diagnoses. This study might also spur positive advancement in the evaluation of facial paralysis and diagnoses of other neuromuscular-inclined diseases.

## Data Availability Statement

The data used to support the findings of this study are available on reasonable request from the corresponding author.

## Ethics Statement

The studies involving human participants were reviewed and approved by Institutional Review Board of Shenzhen Institutes of Advanced Technology, Chinese Academy of Sciences (SIAT-IRB-170815-H0178). The patients/participants provided their written informed consent to participate in this study.

## Author Contributions

SC and GL: conceptualization. MZ and XW: data curation and writing—original draft. MZ, SC, and GL: funding acquisition. MZ and HD: methodology. SC: supervision. XW, YH, and HZ: validation. MZ and ZL: visualization. MZ and MW: writing—review and editing. All authors contributed to the article and approved the submitted version.

## Funding

This work was supported in part by the National Natural Science Foundation of China (#81927804, #62101538, and #61901464), the Shenzhen Governmental Basic Research Grant (#JCYJ20180507182241622), the Science and Technology Planning Project of Shenzhen (#JSGG20210713091808027 and #JSGG20211029095801002), and the SIAT Innovation Program for Excellent Young Researchers (E1G027).

## Conflict of Interest

The authors declare that the research was conducted in the absence of any commercial or financial relationships that could be construed as a potential conflict of interest.

## Publisher's Note

All claims expressed in this article are solely those of the authors and do not necessarily represent those of their affiliated organizations, or those of the publisher, the editors and the reviewers. Any product that may be evaluated in this article, or claim that may be made by its manufacturer, is not guaranteed or endorsed by the publisher.

## References

[B1] Alku P.. (2011). Glottal inverse filtering analysis of human voice production — a review of estimation and parameterization methods of the glottal excitation and their applications. Sadhana 36, 623–650. 10.1007/s12046-011-0041-5

[B2] Balata P. M. M. da Silva H. J. de Araújo Pernambuco L. de Oliveira J. H. P. de Moraes S. R. A. (2015). Normalization patterns of the surface electromyographic signal in the phonation evaluation. J. Voice 29, e121.1–8. 10.1016/j.jvoice.2014.03.010 24930371

[B3] Bishop J. Keating P. (2012). Perception of pitch location within a speaker's range: fundamental frequency, voice quality and speaker sex. J. Acoust. Soc. Am. 132, 1100–1112. 10.1121/1.4714351 22894229

[B4] Bracken D. J. Ornelas G. Coleman T. P. Weissbrod P. A. (2019). High-density surface electromyography: a visualization method of laryngeal muscle activity. Laryngoscope 129, 2347–2353. 10.1002/lary.27784 30663053

[B5] Brajot F.-X. Lawrence D. (2018). Delay-induced low-frequency modulation of the voice during sustained phonation. J. Acoust. Soc. Am. 144, 282–291. 10.1121/1.5046092 30075671

[B6] Chen M. Zhang X. Zhou P. (2018). A novel validation approach for high-density surface EMG decomposition in motor neuron disease. IEEE Trans. Neural Syst. Rehabil. Eng. 26, 1161–1168. 10.1109/TNSRE.2018.2836859 29877840 PMC6317993

[B7] Chhetri D. K. Neubauer J. (2015). Differential roles for the thyroarytenoid and lateral cricoarytenoid muscles in phonation. Laryngoscope 125, 2772–2777. 10.1002/lary.25480 26198167 PMC4715570

[B8] Craig J. Tomlinson C. Stevens K. Kotagal K. Fornadley J. Jacobson B.. (2015). Combining voice therapy and physical therapy: a novel approach to treating muscle tension dysphonia. J. Commun. Disord. 58, 169–178. 10.1016/j.jcomdis.2015.05.001 26012419 PMC4653091

[B9] Cutiva L. C. C. Vogel I. Burdorf A. (2013). Voice disorders in teachers and their associations with work-related factors: a systematic review. J. Commun. Disord. 46, 143–155. 10.1016/j.jcomdis.2013.01.001 23415241

[B10] Dennis J. Tran H. D. Li H. (2010). Spectrogram image feature for sound event classification in mismatched conditions. IEEE Signal Process. Lett. 18, 130–133. 10.1109/LSP.2010.2100380

[B11] Dewan K. Vahabzadeh-Hagh A. Soofer D. Chhetri D. K. (2017). Neuromuscular compensation mechanisms in vocal fold paralysis and paresis. Laryngoscope 127, 1633–1638. 10.1002/lary.26409 28059441 PMC5701745

[B12] Echternach M. Burk F. Köberlein M. Herbst C. T. Döllinger M. Burdumy M.. (2017). Oscillatory characteristics of the vocal folds across the tenor passaggio. J. Voice 31, 381.e5–e381.e14. 10.1016/j.jvoice.2016.06.015 27499033

[B13] Gerratt B. R. Kreiman J. Garellek M. (2016). Comparing measures of voice quality from sustained phonation and continuous speech. J. Speech Lang. Hear. Res. 59, 994–1001. 10.1044/2016_JSLHR-S-15-0307 27626612 PMC5345563

[B14] Glaser V. Holobar A. (2018). Motor unit identification from high-density surface electromyograms in repeated dynamic muscle contractions. IEEE Trans. Neural Syst. Rehabil. Eng. 27, 66–75. 10.1109/TNSRE.2018.2885283 30571641

[B15] Horáček J. Bula V. Radolf V. Vampola T. Duškov,á M. (2016). Development of self-oscillating human vocal folds prosthesis. Proc. Eng. 144, 867–874. 10.1016/j.proeng.2016.05.103

[B16] Hu Y. Kwok J. W. Tse J. Y.-H. Luk K. D.-K. (2014). Time-varying surface electromyography topography as a prognostic tool for chronic low back pain rehabilitation. Spine J. 14, 1049–1056. 10.1016/j.spinee.2013.11.060 24530438

[B17] Jani M. P. Gore G. B. (2014). Occurrence of communication and swallowing problems in neurological disorders: analysis of forty patients. NeuroRehabilitation 35, 719–727. 10.3233/NRE-141165 25318773

[B18] Johns G. Morin E. Hashtrudi-Zaad K. (2016). Force modelling of upper limb biomechanics using ensemble fast orthogonal search on high-density electromyography. IEEE Trans. Neural Syst. Rehabil. Eng. 24, 1041–1050. 10.1109/TNSRE.2016.2515087 26761839

[B19] Kaneko M. Hitomi T. Takekawa T. Tsuji T. Kishimoto Y. Hirano S. (2018). Effects of voice therapy on laryngeal motor units during phonation in chronic superior laryngeal nerve paresis dysphonia. J. Voice 32, 729–733. 10.1016/j.jvoice.2017.08.026 28967588

[B20] Khoddami S. M. Talebian S. Izadi F. Ansari N. N. (2017). Validity and reliability of surface electromyography in the assessment of primary muscle tension dysphonia. J. Voice 31, 386.e9–386.e17. 10.1016/j.jvoice.2016.09.010 27742497

[B21] Kim J. Wigram T. Gold C. (2009). Emotional, motivational and interpersonal responsiveness of children with autism in improvisational music therapy. Autism 13, 389–409. 10.1177/1362361309105660 19535468

[B22] Knuijt S. Kalf J. G. de Swart B. J. Drost G. Hendricks H. T. Geurts A. C.. (2014). Dysarthria and dysphagia are highly prevalent among various types of neuromuscular diseases. Disabil. Rehabil. 36, 1285–1289. 10.3109/09638288.2013.845255 24151818

[B23] Kryshtopava M. Van Lierde K. Meerschman I. D'haeseleer E. De Moor M. Vandemaele P.. (2017). Functional magnetic resonance imaging study of brain activity associated with pitch adaptation during phonation in healthy women without voice disorders. J. Voice 31, 118.e21–e28. 10.1016/j.jvoice.2016.02.022 27049447

[B24] Lolli S. Lewenstein A. D. Basurto J. Winnik S. Loui P. (2015). Sound frequency affects speech emotion perception: results from congenital amusia. Front. Psychol. 6, 1340. 10.3389/fpsyg.2015.01340 26441718 PMC4561757

[B25] Macdonald I. Rubin J. S. Blake E. Hirani S. Epstein R. (2012). An investigation of abdominal muscle recruitment for sustained phonation in 25 healthy singers. J. Voice 26, 815.e19–815.e16. 10.1016/j.jvoice.2012.04.006 23177746

[B26] Martins R. H. G. Pereira E. R. B. N. Hidalgo C. B. Tavares E. L. M. (2014). Voice disorders in teachers. A review. J. Voice 28, 716–724. 10.1016/j.jvoice.2014.02.008 24929935

[B27] Miller A. L. Brugman J. Sands B. Namaseb L. Exter M. Collins C. (2009). Differences in airstream and posterior place of articulation among N| uu clicks. J. Int. Phon. Assoc. 39, 129–161. 10.1017/S002510030900386728161511

[B28] Murtola T. Malinen J. Geneid A. Alku P. (2019). Analysis of phonation onsets in vowel production, using information from glottal area and flow estimate. Speech Commun. 109, 55–65. 10.1016/j.specom.2019.03.007

[B29] Naik G. R. Al-Timemy A. H. Nguyen H. T. (2015). Transradial amputee gesture classification using an optimal number of sEMG sensors: an approach using ICA clustering. IEEE Trans. Neural Syst. Rehabil. Eng. 24, 837–846. 10.1109/TNSRE.2015.2478138 26394431

[B30] Pettersen V. Bjørkøy K. Torp H. Westgaard R. H. (2005). Neck and shoulder muscle activity and thorax movement in singing and speaking tasks with variation in vocal loudness and pitch. J. Voice 19, 623–634. 10.1016/j.jvoice.2004.08.007 16301107

[B31] Phinyomark A. Phukpattaranont P. Limsakul C. (2012). Feature reduction and selection for EMG signal classification. Expert Syst. Appl. 39, 7420–7431. 10.1016/j.eswa.2012.01.102

[B32] Rinaldi L. Lega C. Cattaneo Z. Girelli L. Bernardi N. F. (2016). Grasping the sound: auditory pitch influences size processing in motor planning. J. Exp. Psychol. Hum. Percept. Perform. 42, 11. 10.1037/xhp0000120 26280267

[B33] Rosen C. A. Murry T. (2000). Voice handicap index in singers. J. Voice 14, 370–377. 10.1016/S0892-1997(00)80082-X 11021504

[B34] Sommerville R. B. Vincenti M. G. Winborn K. Casey A. Stitziel N. O. Connolly A. M.. (2017). Diagnosis and management of adult hereditary cardio-neuromuscular disorders: a model for the multidisciplinary care of complex genetic disorders. Trends Cardiovasc. Med. 27, 51–58. 10.1016/j.tcm.2016.06.005 27452966

[B35] Strazzulla I. Nowak M. Controzzi M. Cipriani C. Castellini C. (2016). Online bimanual manipulation using surface electromyography and incremental learning. IEEE Trans. Neural Syst. Rehabil. Eng. 25, 227–234. 10.1109/TNSRE.2016.2554884 28113557

[B36] Tang X. Zhang X. Gao X. Chen X. Zhou P. (2018). A novel interpretation of sample entropy in surface electromyographic examination of complex neuromuscular alternations in subacute and chronic stroke. IEEE Trans. Neural Syst. Rehabil. Eng. 26, 1878–1888. 10.1109/TNSRE.2018.2864317 30106682 PMC6344944

[B37] Van Houtte E. Claeys S. D'haeseleer E. Wuyts F. Van Lierde K. (2013). An examination of surface EMG for the assessment of muscle tension dysphonia. J. Voice 27, 177–186. 10.1016/j.jvoice.2011.06.006 21889301

[B38] Xu X. Yang P. Zhuang P. Yanchao J. Yanli M. Schrof C.. (2018). Study on normal laryngeal electromyography of thyroarytenoid muscle, cricothyroid muscle, and posterior cricoarytenoid muscle. Ann. Otol. Rhinol. Laryngol. 127, 806–811. 10.1177/0003489418796525 30187765

[B39] Zhang M. Wang Y. Wei Z. Yang M. Luo Z. Li G. (2020). Inductive conformal prediction for silent speech recognition. J. Neural Eng. 17:066019. 10.1088/1741-2552/ab7ba0 32120355

[B40] Zhu M. Liang F. Samuel O. W. Chen S. Yang W. Lu L.. (2017a). “A pilot study on the evaluation of normal phonating function based on high-density sEMG topographic maps,” in the 2017 39th Annual International Conference of the IEEE Engineering in Medicine and Biology Society (EMBC), Jeju, 1030–1033. 10.1109/EMBC.2017.8037002 29060049

[B41] Zhu M. Lu L. Yang Z. Wang X. Liu Z. Wei W.. (2018a). “Contraction patterns of neck muscles during phonating by high-density surface electromyography,” in the 2018 IEEE International Conference on Cyborg and Bionic Systems (CBS), Shenzhen, 572–575. 10.1109/CBS.2018.8612181

[B42] Zhu M. Samuel O. W. Yang Z. Lin W. Huang Z. Fang P.. (2018b). “Using muscle synergy to evaluate the neck muscular activities during normal swallowing,” in the 2018 40th Annual International Conference of the IEEE Engineering in Medicine and Biology Society (EMBC), Honolulu, HI, 2454–2457. 10.1109/EMBC.2018.8512760 30440904

[B43] Zhu M. Yu B. Yang W. Jiang Y. Lu L. Huang Z.. (2017b). Evaluation of normal swallowing functions by using dynamic high-density surface electromyography maps. Biomed. Eng. 16, 133. 10.1186/s12938-017-0424-x 29157238 PMC5696778

